# Sinking particles exporting diatoms and Hacrobia predict the magnitude of oceanic POC flux

**DOI:** 10.1093/ismejo/wraf105

**Published:** 2025-05-22

**Authors:** Sasha J Kramer, Erin L Jones, Margaret L Estapa, Nicola L Paul, Tatiana A Rynearson, Alyson E Santoro, Sebastian Sudek, Colleen A Durkin

**Affiliations:** Monterey Bay Aquarium Research Institute, 7700 Sandholdt Rd, Moss Landing, CA, 95039, United States; Graduate School of Oceanography, University of Rhode Island, 215 S. Ferry Rd, Narragansett, RI, 02882, United States; School of Marine Sciences, Darling Marine Center, University of Maine, 193 Clarks Cove Rd, Walpole, ME, 04573, United States; Department of Ecology, Evolution, and Marine Biology, University of California, Santa Barbara, CA, 93117, United States; Graduate School of Oceanography, University of Rhode Island, 215 S. Ferry Rd, Narragansett, RI, 02882, United States; Department of Ecology, Evolution, and Marine Biology, University of California, Santa Barbara, CA, 93117, United States; Monterey Bay Aquarium Research Institute, 7700 Sandholdt Rd, Moss Landing, CA, 95039, United States; Monterey Bay Aquarium Research Institute, 7700 Sandholdt Rd, Moss Landing, CA, 95039, United States

**Keywords:** carbon export, phytoplankton, diatoms, biological pump

## Abstract

Carbon flux to the deep sea can be dictated by surface ocean phytoplankton community composition, but translating surface ocean observations into quantitative predictions of carbon export requires additional consideration of the underlying ecosystem drivers. Here, we used genetic tracers of phytoplankton detected in surface seawater and within sinking particles collected in the mesopelagic ocean to identify mechanistic links between surface communities and carbon export in the North Pacific and North Atlantic Oceans. Phytoplankton 18S rRNA gene sequences were sampled over a 1-month period in surface seawater and within bulk-collected and individually isolated sinking particles using mesopelagic sediment traps (100–500 m). Nearly all phytoplankton amplicon sequence variants exported from the surface were packaged in large (>300 μm) particles. Individually, each of these particles contained only a few distinct phytoplankton amplicon sequence variants, but collectively, large particles transported about half of the surface taxonomic diversity into the mesopelagic. The relative sequence abundances of the surface community detected within particles were quantitatively related to measured carbon fluxes: a linear model based on the relative sequence abundance of just two pigment-based phytoplankton taxa, diatoms and photosynthetic Hacrobia, was predictive of carbon flux magnitude. These two taxa were also enriched in the ecologically distinct particle classes that had the greatest influence on carbon export magnitude. As global, hyperspectral ocean color satellites begin to quantify these taxonomic groups in the surface ocean, the relationship of these taxa to carbon fluxes demonstrated here may help in developing more accurate algorithms to estimate global carbon export in the ocean.

## Introduction

Each year, ~10 Pg of carbon are exported from the surface ocean to the deep sea [1]. Best estimates of this component of the global carbon cycle rely on measurements from ocean color satellites that collect synoptic observations of surface ocean phytoplankton communities [2]. Further models are required to estimate how much of this primary production is exported from the surface ocean in the form of sinking particles and eventually transported into the deep ocean and sequestered for climate-relevant timescales [3, 4]. These models must predict two separate processes of the biological carbon pump [5] to calculate deep ocean carbon sequestration. First, how much of the surface biomass is exported below the euphotic zone? And second, how much of the exported biomass is transferred through the mesopelagic ocean as particles sink? The biological mechanisms that control these export processes are poorly constrained, making the biological carbon pump among the most uncertain components of the global carbon cycle.

Some key processes influencing the biological carbon pump are well described [4], but translating these observations into relationships that are useful for the accurate prediction of carbon export remains a challenge. For example, observations of surface ocean phytoplankton community composition have improved due to investment in global surveys (e.g. Tara Oceans, Bio-GO-SHIP; [6, 7]) and in technologies that increase taxonomic resolution (such as deoxyribonucleic acid [DNA] sequencing; [8]) and spatial resolution (such as NASA’s Plankton, Aerosol, Cloud, ocean Ecosystem satellite, PACE; [9]). Collecting these observations is critical, as both the abundance and the taxonomic composition of the surface ocean phytoplankton community can alter how much carbon sinks below the surface ocean and the efficiency of its transfer to the deep sea [10, 11]. Several studies have identified specific phytoplankton taxa that influence carbon export, such as diatoms and coccolithophores (e.g. [12–16]) or networks of plankton communities associated with elevated carbon flux [17]. Other studies have focused on the particles sinking below the surface layer, finding that particles with different ecological origins play distinct roles in packaging surface phytoplankton and transporting carbon into the deep ocean [4, 11]. For example, salp swarms are patchy and episodic, but when salps are present, their fecal pellets efficiently export surface carbon into the deep ocean (e.g. [18]). Collectively, most studies support a key conclusion about the function of the biological carbon pump: not all phytoplankton taxa or particle types are exported with equal efficiency due to variations in cell size and physiology, particle morphology, varying mineral composition, and complex ecosystem interactions such as zooplankton grazing preferences [19, 20]. We must translate these observations into quantitative mechanisms that are useful for predicting both the magnitude of carbon export in the surface ocean and the efficiency of its transfer to sequestration depths.

One way to evaluate contributions of different phytoplankton groups to carbon export is to trace their presence in sinking particles in the mesopelagic [20–22]. DNA and ribonucleic acid [RNA] sequencing have been used to detect phytoplankton communities within sinking particles captured by sediment traps. The addition of particle imagery with sediment gel traps allows for further characterization of particle types alongside genetic characterization of the exported plankton community [20, 23, 24]. Genetic material can be delivered by intact phytoplankton cells or detrital particles, such as zooplankton fecal pellets (e.g. [25–28]). The application of these methods has highlighted the differential contributions of specific phytoplankton taxa to particulate organic carbon (POC) export flux depending on the region and season, as well as the zooplankton grazers that are present to facilitate that export [24, 29–31]. Often, high export flux events are comprised of particles containing one or a few dominant taxa [27, 30]. Despite these advances, work remains to quantify the predictive power of specific phytoplankton taxa for estimating POC flux into the mesopelagic across the regional and seasonal scales needed to make global estimates, and with the taxonomic resolution achievable from space [32, 33].

Here, we investigated the distribution of eukaryotic, photosynthetic phytoplankton from the surface ocean to the mesopelagic, from whole seawater to sinking particles [20, 23, 34]. Sampling was conducted as part of the EXport Processes in the Ocean from RemoTe Sensing (EXPORTS) field campaigns aboard R/V *Roger Revelle* in the eastern North Pacific Ocean (Ocean Station Papa, August–September 2018; [35]) and aboard RRS *James Cook* in the eastern North Atlantic Ocean (Porcupine Abyssal Plain, May 2021; [36]). Sampling included sediment trap deployments and whole seawater sample collection. POC flux magnitude was higher in the North Atlantic, driven by pulses of fresh phytodetritus and intense storm activity [37–39]. In the North Pacific, a highly recycled ecosystem was sampled, with lower POC flux magnitude that was at times enhanced by salp grazing activity [18, 39–41]. We resolved the genetic contents of 770 individually isolated particles from the mesopelagic, which allowed us to identify distinct export mechanisms of surface phytoplankton groups relevant for the development of predictive export models. First, we found that most phytoplankton were exported out of the surface ocean in large (>300 μm) particles. Second, particles produced by different ecological processes exported distinct components of the phytoplankton community. Finally, when those particles were enriched in diatoms and photosynthetic Hacrobia, more carbon was exported to the deep ocean—thus, the relative abundance of these taxa within sinking particles can be used to model POC flux.

## Materials and methods

### Sediment trap sampling

On each cruise, sediment traps were deployed three times, for a duration between 2 to 6 days. Details of sediment trap deployments are found in [40, 42]. DNA and POC samples described here were collected from both Surface-Tethered Traps (STTs; [43]) and Neutrally Buoyant Sediment Traps (NBSTs; [44]). The STT array collected sinking particles at five depths while the NBSTs were deployed at a subset of those five depths. The shallowest traps were deployed below the euphotic zone, defined by the 1% surface light level. Traps were deployed in three successive collection periods over one month. Additional details about sediment trap preparation and deployment are found in the Supporting Information.

### Bulk sediment trap samples

Bulk particle collections (North Pacific *n* = 35; North Atlantic *n* = 17) were sampled for nucleic acids following [45]. Particles from one collection tube were filtered with vacuum filtration onto a 0.2 μm Supor filter, placed in cryovials, flash frozen in liquid nitrogen, and stored at −80°C. Total DNA was extracted using the AllPrep PowerViral DNA/RNA extraction kit (Qiagen, Hilden, Germany) following manufacturer’s instructions. These samples are labeled “bulk particles” in this work.

### Individual particles

Imaging of particles collected in polyacrylamide gels is described in [23] and the collection of nucleic acids from individual sinking particles is described in [20, 45]. Polyacrylamide gels were removed from the trap tubes upon recovery and quantitatively imaged at multiple magnifications using a stereomicroscope (Olympus SZX16 with Lumenera Infinity 2 camera). Imaged particles were quantified, sized, and classified using Python-based image processing scripts, and their contributions to POC flux were modeled following [23, 34]. After imaging, particles larger than ~300 μm (North Pacific *n* = 320, North Atlantic *n* = 450) were isolated from the gel using a 1000 μl micropipette and transferred into a cryovial, where they were flash frozen in liquid nitrogen and stored at −80°C until analysis. Four major particle categories were separated via imaging: (i) salp fecal pellets, (ii) aggregates and dense detritus, (iii) long fecal pellets and large loose pellets, and (iv) short pellets (Fig. S1). Individual particle DNA was extracted in a 5%–10% solution of Chelex 100 resin (Bio-Rad, Hercules, CA, USA) by iteratively incubating (at 95°C) and vortexing the sample until solid debris was concentrated at the bottom of the sample tube via centrifugation. The overlying liquid containing nucleic acids was removed [20]. The DNA was further purified and concentrated using the DNA Clean and Concentrator kit (Zymo Research, Orange, CA, USA).

### Euphotic zone seawater sampling

Whole seawater samples were collected in Niskin bottles from three depths (surface, 0.1% light, and 0.01% light) at noon during days spanning the duration of the trap deployments in both the North Pacific and North Atlantic. Samples were filtered onto 0.2 μm pore size, 47 mm polyethersulfone filters (Express Plus; Millipore), flash frozen in liquid nitrogen, and stored at −80°C until processing. Total DNA was extracted from plankton biomass samples (North Pacific *n* = 54, North Atlantic *n* = 54) using the Quick DNA/RNA Miniprep Plus Kit (Cat. No. D7003; ZymoResearch) following manufacturer’s instructions for tissue extraction with the following modifications: 150 μl 0.4 mm Zirconium beads, 60 μl PK Digestion Buffer, and 30 μl Proteinase K were added prior to homogenization (30 s), incubation (2 hours at 55°C), vortexing, and centrifugation to pellet debris (2 min maximum speed).

### Analysis of DNA sequences

Details of the 18S rRNA gene V4 region amplification and sequence analysis are found in the Supporting Information. Processing of 18S V4 rRNA gene sequence reads for all sample types (seawater, bulk traps, and individual particles) was conducted as described in [23] using QIIME2 [46] and the DADA2 [47] workflow. Taxonomy was assigned to amplicon sequence variants (ASVs) using a naïve Bayesian classifier algorithm [48] with a minimum bootstrap confidence of 80% for the Protist Ribosomal Reference ([49]; PR2 v 4.14.0, downloaded September 2022). ASVs were assigned as either photosynthetic or heterotrophic taxa in accordance with prior trophic classification by [20] and with supplemental photosynthetic taxa unique to this dataset. Our analysis focused on photosynthetic eukaryotic phytoplankton ASVs. Dinoflagellate trophic strategies are variable and often fluid, so we included all dinoflagellates in our analysis of phytoplankton while acknowledging that some taxa may be heterotrophic. Known parasitic dinoflagellate taxa (e.g. *Syndiniales* spp.; [50]) were removed. Based on ASV accumulation curves, samples reached saturation at 5000 photosynthetic reads per sample. Thus, we removed nine samples with <5000 reads from further analysis. The code for these analysis steps, including the list of photosynthetic taxa (Table S1), is available on GitHub (see Data Availability Statement).

Phytoplankton ASV presence and relative abundance were compared across sample types. Taxa identified in both the individually picked particles and corresponding bulk sediment trap samples from the same trap platform were considered to be packaged within “large” (> 300 μm) particles. Taxa found in the bulk sediment trap samples but not in the individual particles isolated from the same trap platform were assumed to be packaged in particles smaller than could be isolated for successful DNA amplification (<300 μm).

Relative phytoplankton read counts were center log-ratio transformed, and sample composition was compared using a compositional principal components analysis (PCA) in Matlab v. R2022a [51]. The singular value decomposition (SVD) approach was used to construct the PCA (“svd” function in Matlab), and photosynthetic phytoplankton sequences were grouped to the genus-level for this analysis (*n* = 132). Differences among sample groups were assessed from Aitchison distances [52] using PERMANOVA with Bonferroni p-value adjustment for multiple comparisons. PCA was also used to examine the co-variability in phytoplankton groups and POC flux. For this analysis, SVD was not used to calculate the PCA (“pca” function in Matlab). Variables were mean-centered and normalized by their standard deviation before PCA was performed.

### Relating phytoplankton community composition from DNA to pigment-based methods

For all further analyses, phytoplankton taxa from 18S rRNA genes were divided into six pigment-based taxonomic groups (following [53]) to enable applications of these results to satellite-derived estimates of phytoplankton community composition in the future [32]. Phytoplankton pigment concentrations were measured by high-performance liquid chromatography (HPLC) from both EXPORTS field campaigns. The sum of the accessory pigments in this dataset was correlated with total chlorophyll-*a* concentration (*R^2^* = 0.98). The phytoplankton groups and their assumed corresponding accessory pigments are as follows: diatoms (fucoxanthin), dinoflagellates (peridinin), photosynthetic Hacrobia (prymnesiophytes [19’hexanoyloxyfucoxanthin] and cryptophytes [alloxanthin]), Chlorophytes (monovinyl chlorophyll b), Dictyochophytes and Pelagophytes (19’butanoyloxyfucoxanthin), and other Ochrophyta (20% of total chlorophyll c, assuming an equal distribution across all red algal classes shown here). As found in [54], relative accessory pigment concentrations to total chlorophyll-*a* and relative 18S rRNA gene sequence abundances were positively correlated in surface ocean samples for the major relevant groups in this dataset (diatoms: *R^2^* = 0.65, *P* value < .001; Hacrobia: *R^2^* = 0.41, *P* value < .001; all other summed photosynthetic phytoplankton: *R^2^* = 0.61, *P* value < .001).

## Results

### Sinking particles contained a distinct phytoplankton community

**Figure 1:**
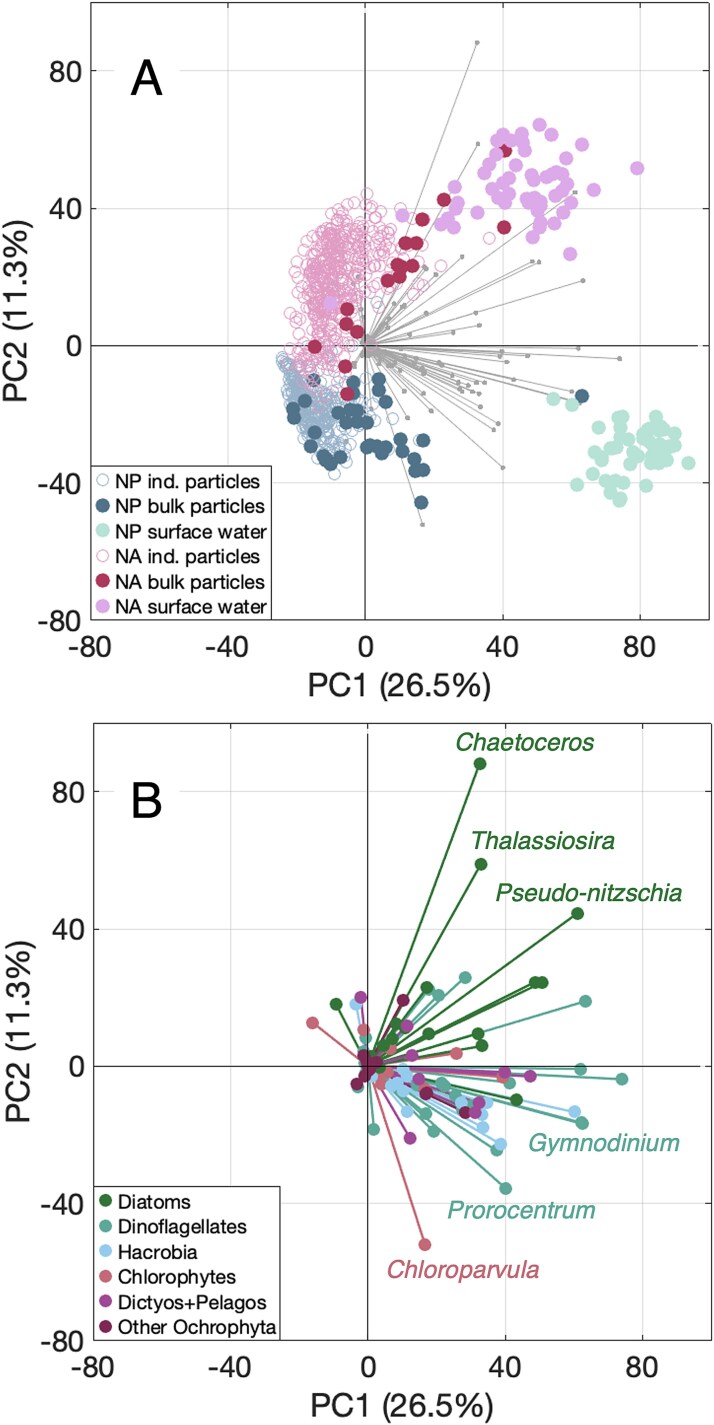
Differences in phytoplankton community composition among surface seawater and sinking particles. PCA plot showing the first two components for all surface, bulk trap, and particle samples in both basins [(A) points are shown in relation to the sample type and the basin in which they were collected, as indicated by the legend on the bottom left] and genus-level ASV bins driving this variability [vectors in (A) are colored based on the phytoplankton group indicated by the legend on the bottom left in (B)]. A subset of the genera with the largest loadings on each axis are labeled in (B). Dictyos + Pelagos refers to Dictyochophytes and Pelagophytes.

Phytoplankton communities differed by ocean basin, depth, and collection method based on 18S rRNA gene relative abundances. All North Pacific samples differed significantly from all North Atlantic samples (Fig. 1A shows the PCA of phytoplankton community composition across all sample types; PERMANOVA of Aitchison distances computed from PCA; adjusted *P* value = .006). North Atlantic samples were positively associated with diatoms, especially the genera *Chaetoceros*, *Thalassiosira*, and *Pseudo-nitzschia*. North Pacific samples were positively associated with dinoflagellates (*Gymnodinium*, *Gyrodinium*, *Prorocentrum*), Hacrobia (*Phaeocystis* [prymnesiophyte], *Chrysochromulina* [prymnesiophyte], *Plagioselmis* [cryptophyte]), and chlorophytes (*Chloroparvula*, *Bathycoccus*; Fig. 1B highlights the genus-level ASV bins). The largest variability among all samples was between surface seawater communities and all other sample types, which separated on the first principal component (26.5% of variability; Fig. 1A; PERMANOVA of Aitchison distances; NP adjusted *P* value = .003; NA adjusted *P* value = .003). Communities found in individual particles also differed from those found in bulk particle samples (PERMANOVA of Aitchison distances; NP adjusted *P* value = .0028; NA adjusted *P* value = .0021).

**Figure 2:**
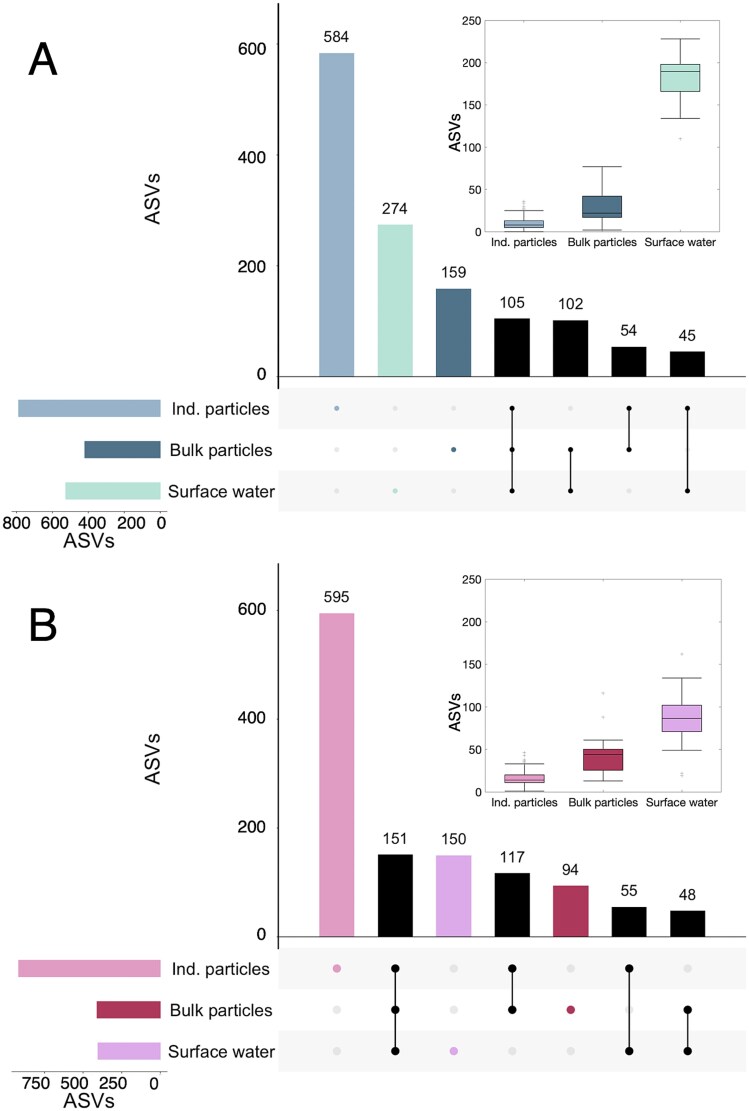
Number of phytoplankton ASVs shared among three sample types in (A) the North Pacific and (B) the North Atlantic: Individual (ind.) particles, bulk particles, and surface seawater. Bars in the upper panel show the number of unique or shared ASVs in each sample category or grouping (indicated by the dots in the lower panel). Bars on the left panel show the total number of ASVs in that sample category. Inset boxplots show the average number of ASVs per sample across all samples in each category (center line) and the range of values to the 25th and 75th percentiles, respectively (edges of the box). Whiskers cover +/−2.7 times the standard deviation of the data, and outliers from this range are shown as crosses.

Despite their differing compositions, samples of the same type from different ocean basins shared similar alpha diversity of phytoplankton (ASV richness; Fig. 2 [55]). The fraction of ASVs shared among all three sample types (surface seawater, bulk particles, and individual particles) was small (Fig. 2A, North Pacific = 105 ASVs, 8% of total; Fig. 2B, North Atlantic = 151 ASVs, 12% of total). The surface seawater communities in the North Pacific were more diverse (526 ASVs) than in the North Atlantic (410 ASVs). A greater proportion of that diversity was only found in the surface seawater (274 ASVs; 21% of total) compared to the North Atlantic (150 ASVs; 12% of total). In contrast to the average surface seawater sample, the average diversity within individual particles was low (Fig. 2, inset boxplots), with a slightly higher per-particle diversity detected in the North Atlantic (Fig. 2B, median 14 ASVs per particle) than the North Pacific (Fig. 2A, median 8 ASVs per particle). However, when considered all together, the particle pool contained the greatest fraction of total diversity, most of which was not shared with other sample types. In the North Pacific, individual particles comprised 584 ASVs (or 44% of the total) that were not found in the other pools. In the North Atlantic, individual particles comprised 595 ASVs (or 49% of the total) that were not found in other pools. In each basin, the mean ASV diversity in each sample type differed significantly from the other two sample types (one-way ANOVA with Tukey–Kramer post hoc; NP *P* < .001; NA *P* < .001). Each individual particle transported a small and highly variable subset of the total phytoplankton diversity found in the bulk sinking particles and the surface seawater. The bulk sinking particle communities had the lowest number of unique ASVs (Fig. 2A, 159 or 12% in the North Pacific; Fig. 2B, 94% or 7% in the North Atlantic).

### Large particles transported most exported phytoplankton from the surface ocean

**Figure 3:**
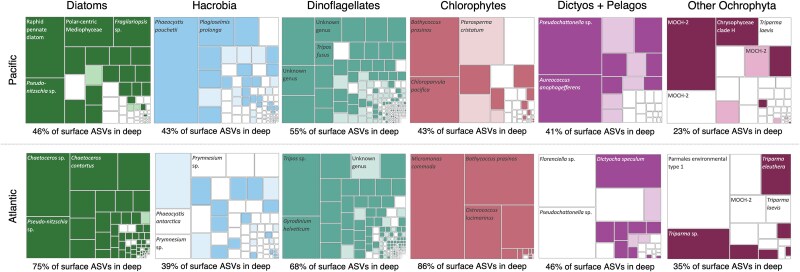
Identification of phytoplankton taxa exported from surface seawater in the North Pacific (top row) and the North Atlantic (bottom row). Relative abundances of 18S rRNA gene sequences in surface seawater were grouped by major pigment classifications. Each small box within a larger box represents an individual ASV within that class, scaled to the fraction of reads. If the box is colored in, that ASV was found in a sediment trap sample (darker color = in bulk particles and individual particles, lighter color = only in bulk particles, not individual particles). Boxes that are not colored in represent ASVs that were not found in sinking particles. Dictyos + Pelagos refers to Dictyochophytes and Pelagophytes.

**Figure 4:**
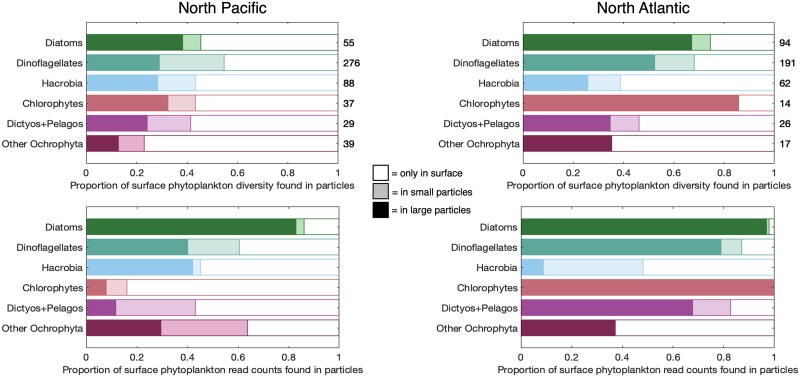
Proportion of the surface ocean phytoplankton 18S rRNA gene diversity (top panels) and read counts (bottom panels) that were exported in the North Pacific (left) and North Atlantic (right). Phytoplankton 18S rRNA gene sequence read counts were grouped based on major pigment classes. Colored bars represent the proportion of ASVs in the surface that were also found in a sediment trap sample (darker color = in bulk particles and individual (ind.) particles, lighter color = only in bulk particles, not individual particles). White bars represent the proportion of ASVs that were found on the surface but not in sinking particles. The total number of 18S rRNA gene ASVs in the surface ocean for each phytoplankton group is enumerated at the end of the bar for the top panels. Dictyos + Pelagos refers to Dictyochophytes and Pelagophytes.

In the North Pacific, a little less than half of the surface ASV diversity was detected in sinking particles on average, whereas in the North Atlantic, more than half of the surface phytoplankton ASV diversity was also found in sinking particles (summed bulk and individual particles; Figs 3 and 4). In both ocean ecosystems, most exported phytoplankton ASVs from the surface were detected in large (>300 μm), individual sinking particles rather than only in bulk-collected particle samples (Fig. 3). Large particles exported the largest fraction of ASV diversity (Fig. 4) and the ASVs with the largest fraction of relative read counts in the surface (Fig. 3), excluding chlorophyte reads in the North Pacific. A similar link between large particles and surface phytoplankton was identified when the comparison was reversed (all phytoplankton ASVs in sinking particles vs. those shared in the surface seawater, Fig. S2). The majority of the ASVs found in bulk sinking particles were also shared with individual particles (Fig. S3), further demonstrating the dominance of larger particles for packaging phytoplankton ASVs.

### Large particle classes packaged distinct communities of phytoplankton

**Figure 5:**
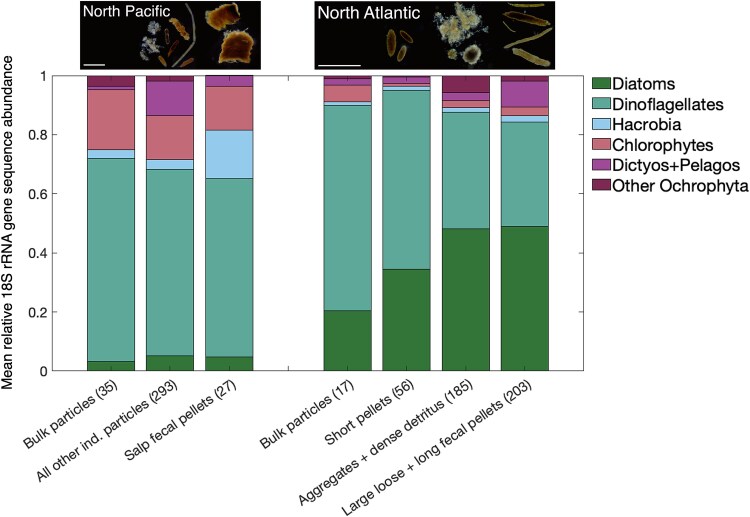
Particle types containing significantly different communities of phytoplankton 18S rRNA gene sequences in the North Atlantic and North Pacific. Significant differences in phytoplankton ASV community composition were identified among all sample replicates (replicate numbers indicated on x-axis). The figure displays the average community composition of all particle replicates and phytoplankton taxa grouped into major pigment-based classes. Representative dark-field microscopy images of major particle types are shown above the corresponding bar for each basin (white scale bar = 1 mm). Dictyos + Pelagos refers to Dictyochophytes and Pelagophytes.

We next investigated whether large particles of differing ecological origin transported distinct subsets of the phytoplankton community from the surface ocean to the deep ocean. As noted earlier, the ASV diversity was low and highly variable among individual particles. Still, salp fecal pellets contained a distinct community from all other North Pacific particles, with higher relative Hacrobia sequence reads (specifically, one *Chrysochromulina* spp. ASV) on average than other particle types (Fig. S4; PERMANOVA of Aitchison distances; adjusted *P* value = .0028). In the North Atlantic, three ecologically distinct particle classes contained different sequence compositions: aggregates/dense detritus, large-loose/long fecal pellets, and short pellets (PERMANOVA of Aitchison distances; adjusted *P* value = .0021). The relative diatom sequence abundance was 15%–25% higher on average in the first two particle categories compared to short fecal pellets (Fig. 5). Both aggregates and long fecal pellets contained reads of *Chaetoceros* spp., but aggregates also contained relatively more *Thalassiosira* spp. reads.

### Enrichment of diatoms and photosynthetic Hacrobia in sinking particles is predictive of POC flux magnitude

**Figure 6:**
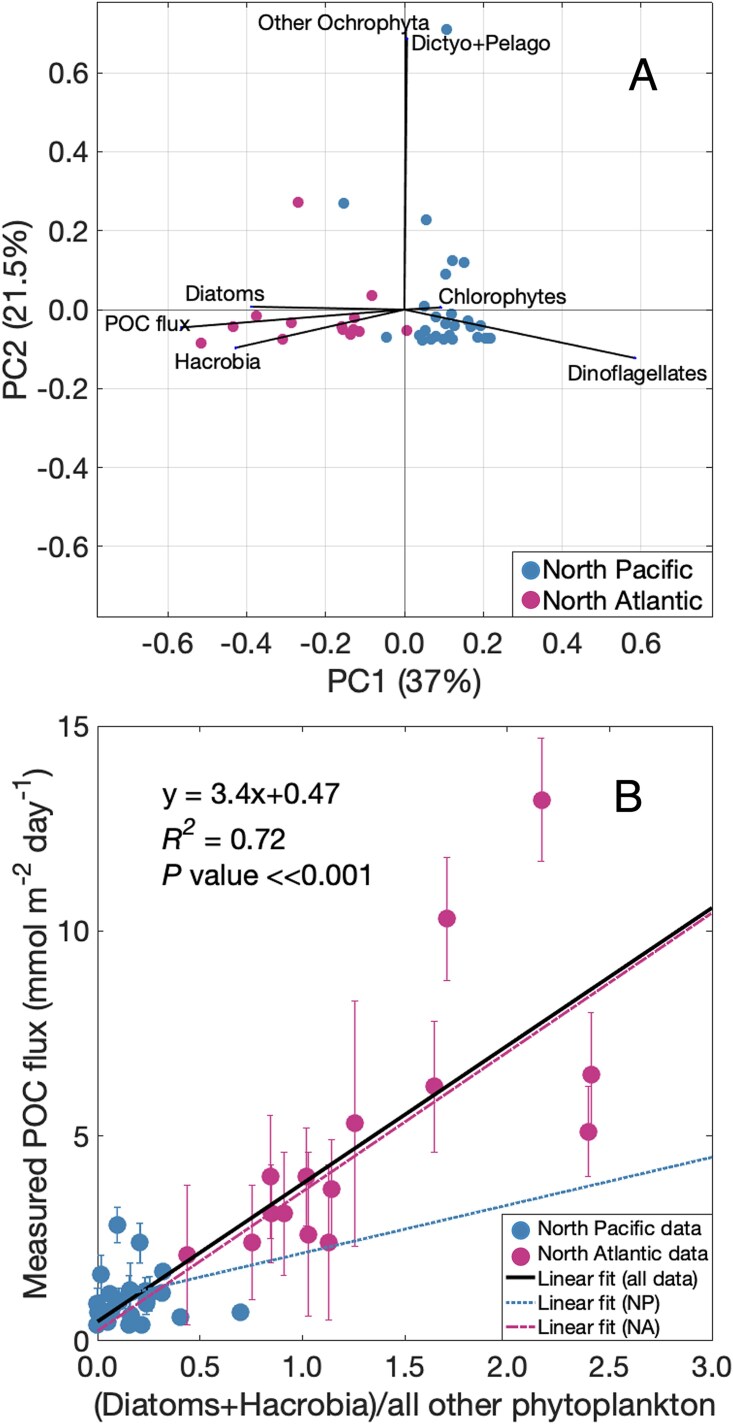
Relationship between phytoplankton communities within sinking particles and POC fluxes. (A) PCA of pigment-based phytoplankton groups obtained from ASV data in bulk sediment traps. The orientation of each sample and each variable (vectors) is shown for the first two components. (B) Relationship between POC flux and the ratio of diatoms + Hacrobia to all other photosynthetic eukaryotic phytoplankton detected within bulk sinking particles. Samples are colored by ocean basin in both panels, as indicated by the legend at the bottom right of each panel. Error bars represent the uncertainty associated with each POC measurement. The linear fit for all data is shown in a solid line, for NP samples in a dotted line, and for NA samples in a dashed line, also indicated by the legend at the bottom right of panel B.

The relative sequence abundance of specific taxa in the large sinking individual particles was directly related to variability in POC flux by examining bulk particle sample DNA communities using PCA (Fig. 6A) at varying taxonomic resolution (Fig. S5). The first component (37.0% of variability) was most strongly negatively correlated with POC flux (*R^2^* = 0.83) and the relative sequence abundances of diatoms (*R^2^* = 0.38) and Hacrobia (*R^2^* = 0.48), and positively correlated with the relative sequence abundance of dinoflagellates (*R^2^* = 0.88). The second PCA component (21.5% of variability) was strongly positively correlated with the relative sequence abundances of Dictyochophytes + Pelagophytes (*R^2^* = 0.71) and other Ochrophyta (*R^2^* = 0.76). Twenty-six of 28 North Pacific samples were associated with a positive Component 1 (higher relative sequence abundances of dinoflagellates), and 14 of 15 North Atlantic samples had negative loadings on Component 1 (higher relative sequence abundances of diatoms and Hacrobia; higher POC flux). This association of diatoms and photosynthetic Hacrobia with higher POC flux was similarly detected among summed individual particle types and their class-specific contributions to POC flux (Fig. S6; [34]).

This analysis was repeated with ASV-level taxonomic information from the bulk particle samples. In the North Pacific, the ASVs driving most of the variability in POC flux in the bulk trap samples included 2 *Fragilariopsis* spp. ASVs, a *Prymnesiophyceae* ASV, and multiple unidentified dinoflagellate ASVs (Fig. S5). These were different ASVs than those identified as the most relatively abundant in individual particles. In the North Atlantic, the most important driver of POC flux in bulk traps was a *Thalassiosira* spp. ASV, 2 *Pseudo-nitzschia* spp. ASVs, and a *Prymnesiophyceae* ASV. The *Thalassiosira* spp. ASV was one of the most relatively abundant taxa that differed between individual particle types (Fig. S4).

The correlation identified in the PCA led us to test a simple POC flux model based on the sum of diatom and Hacrobia relative sequence abundances normalized to the relative abundance of all other phytoplankton sequences detected in bulk sinking particles. In the North Atlantic, sinking particles exported more total POC and contained greater relative sequence abundances of diatoms and Hacrobia relative to all other phytoplankton, whereas in the North Pacific, the POC flux was lower, with corresponding higher relative abundances of other phytoplankton in sinking particles relative to diatoms and Hacrobia (Fig. 6B).

We calculated a linear relationship (y = 3.4x + 0.47, where x = [diatoms + Hacrobia]/all other phytoplankton; *R^2^* = 0.72, *P* < .001) using the relative abundance of only ASVs that were shared between sinking particles and surface seawater (Figs 6B and S2). No significant relationship was found when performing this analysis on all phytoplankton taxa detected in particles, including those ASVs not found in surface ocean samples. This relationship was based on basin-scale variations: there was no independent linear relationship found between these variables in only the North Pacific samples (y = 0.17x + 0.97; *R^2^* = 0.002, *P* value = .83) and only a weak relationship found in the North Atlantic (y = 3.4x + 0.24; *R^2^* = 0.48*, P* value = .003). Relationships were also tested for only diatoms/all other phytoplankton and only Hacrobia/all other phytoplankton in both basins and across the entire dataset (Table S2), but the strongest relationship with POC flux was consistently found with [diatoms + Hacrobia]/all other phytoplankton data from both basins.

**Figure 7:**
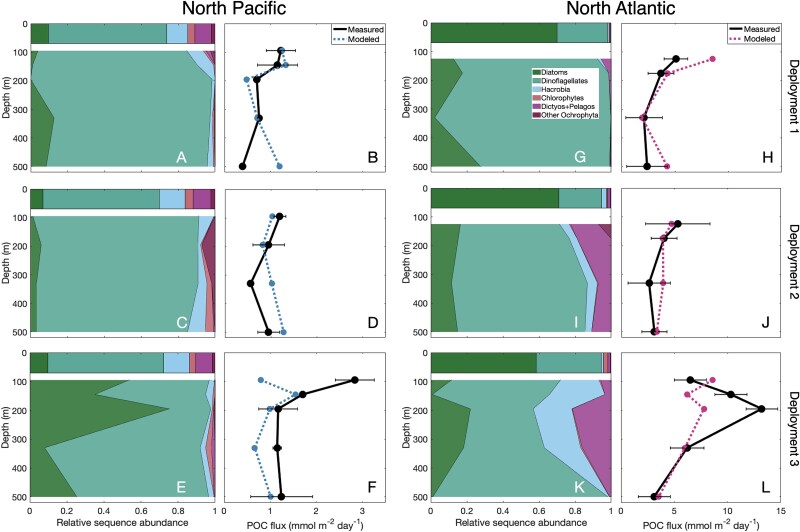
Site-specific prediction of POC flux in the top 500 m based on phytoplankton 18S rRNA gene sequences (A, C, E, G, I, K). Measured POC flux and modeled POC flux (B, D, F, H, J, L) from the (diatoms + Hacrobia) / all other phytoplankton ratio (by applying the equation in Fig. 6B), all from bulk particle samples, at each collection depth during three separate sediment trap deployments in the North Pacific (left) and the North Atlantic (right). All samples shown here were collected from STTs to maximize match-ups between POC and 18S rRNA gene samples across depths and deployments. Dictyos + Pelagos refers to Dictyochophytes and Pelagophytes.

Although the linear relationship was based on basin-scale variation, it still adequately represented the spatial and temporal variability in POC flux detected at each site over the course of one month (Fig. 7). In the North Pacific, dinoflagellate reads dominated the phytoplankton community within sinking particles, comprising 62%–98% of sequence reads in Deployment 1 and 2 (Fig. 7A, C). In Deployment 3, however, the relative abundance of diatom reads increased at 95 m and 195 m (Fig. 7E), corresponding to higher POC flux during that time (Fig. 7F). POC flux was typically low in the North Pacific (0.5–1.5 mmol m^−2^ day^−1^), except for the 95 m trap in Deployment 3 (2.8 mmol m^−2^ day^−1^; Fig. 7B, D, F). To compare measured POC flux to flux predicted using DNA sequence data, we used the line of best fit to model POC flux from the ratio of phytoplankton relative sequence abundances. The modeled values varied over the 3 deployments in the North Pacific and broadly mirrored the shape and magnitude of the measured POC flux depth profile to 500 m.

In the North Atlantic, the surface seawater contained relatively higher diatom sequence reads than the North Pacific, comprising 70% of the reads in Deployment 1 (Fig. 7G), 71% in Deployment 2 (Fig. 7I), and 59% in Deployment 3 (Fig. 7K). There were also higher relative sequence abundances of Hacrobia, Dictyochophytes, and Pelagophytes, particularly in Deployments 2 and 3 (Fig. 7I, 7K). These phytoplankton communities corresponded to higher POC flux values that increased from Deployments 1 and 2 (2.1–5.3 mmol m^−2^ day^−1^; Fig. 7H, 7J) to Deployment 3 (6.2–13.2 mmol m^−2^ day^−1^; Fig. 7L). The modeled values again demonstrated the relationship between the phytoplankton community (in this case, higher relative sequence abundances of diatoms and Hacrobia) and higher POC flux magnitudes (Fig. 7H–L).

## Discussion

By probing the contents of hundreds of individually isolated sinking particles, we identified key mechanisms that export phytoplankton from the surface ocean and control the efficiency of their transport into the deep sea. First, we found that most of the eukaryotic, photosynthetic phytoplankton that left the surface ocean were packaged in large sinking particles. The ecological source of these particles also determined which fraction of the surface community was exported: salp fecal pellets exported a distinct surface community enriched in Hacrobia in the North Pacific, whereas aggregates and long fecal pellets each exported distinct communities enriched in different diatom taxa in the North Atlantic. The relative sequence abundance of these two taxa, diatoms and photosynthetic Hacrobia, in bulk particle samples was quantitatively related to the magnitude of POC flux across ocean basins, over time, and over depth. In summary, the relative abundance of diatoms and photosynthetic Hacrobia packaged into large particles predicted the amount of carbon exported out of the surface ocean and its transfer to depth. Further, the ecological source of that large particle determined exactly which surface taxa were selectively exported.

Integrating DNA sequencing data into traditional frameworks of the biological carbon pump can empower future ocean carbon models with direct observations of export mechanisms. The surface phytoplankton community could be traced into sinking POC through the DNA signatures packaged within the particles. The relative abundance of diatoms and photosynthetic Hacrobia 18S rRNA gene sequences within sinking particles was quantitatively related to the absolute magnitude of POC exported by those particles. Although these results support previous studies that identified links between diatoms and some Hacrobia taxa (specifically prymnesiophytes such as coccolithophores) and POC flux (e.g. [14, 17, 56, 57]), we did not expect a measurement that is explicitly relative (amplicon sequence counts) to correlate with a quantitative chemical measurement. We anticipate that this correlation will need to be tested across other ecosystems to consider the applicability beyond our study sites, particularly given the variability in this relationship between ocean basins. Additionally, this relationship was only detectable when considering the ASVs shared between the surface and the sinking particles, suggesting that phytoplankton taxa detectable in surface waters are linked to the corresponding POC fluxes below the surface. Sinking particles also contained ASVs not detected in the surface, but these taxa were not quantitatively related to POC flux magnitude. Diatoms and Hacrobia in the surface are generally associated with “new production,” or growth on nitrate, which must be balanced by carbon export to keep the system in steady state [58]. Our data show that these taxa associated with adding additional carbon into the system were also directly packaged into the exported carbon particles that kept the system in balance through export. We found that most of this export occurred in large sinking particles, no matter whether the system was productive with high-magnitude POC fluxes (i.e. North Atlantic; [59]), or growth-limited with low-magnitude POC fluxes (i.e. North Pacific; [23, 40]). Although our study was limited in geographic range to two ocean basins and in time to two individual months of sampling, we found that the trends we observed between diatoms and Hacrobia and POC export flux magnitude were evident across depths and multiple trap deployments. DNA signatures of phytoplankton within individually isolated particles also connected the large sinking particles with specific ecologically driven export mechanisms, such as grazing by salps or direct aggregation of a diatom bloom [18, 59]. These types of observations could validate and constrain mechanistic models of the biological carbon pump in a way not previously possible.

Our quantitative model relates phytoplankton communities within particles to the magnitude of POC flux throughout the mesopelagic. Our ultimate goal is to better predict POC flux out of the surface ocean based solely on observations of the surface phytoplankton community, but this approach will require an additional transfer function that translates surface phytoplankton biomass and diversity to packaging into sinking particles, going beyond the correlative relationship found here between phytoplankton community composition and quantitative carbon export flux. The results presented here enable us to predict POC flux based on whether diatoms and Hacrobia are packaged into those particles. Identifying this initial transfer function will first require spatially resolved observations of surface ocean phytoplankton community diversity and biomass. This challenge is now being met by the PACE satellite and its Ocean Color Instrument (OCI). Unlike earlier ocean color satellites and associated models, it will be possible to observe phytoplankton community composition via pigment-based phytoplankton groups on unprecedented spatial and temporal scales [32, 33]. Hyperspectral remote sensing observations collected by the OCI allow for the development of new algorithms that utilize information across the visible spectrum of light to model phytoplankton pigment concentrations [32, 60] and link those pigments with phytoplankton taxonomy from higher-resolution methods like HPLC and DNA [54, 61]. Further linking satellite observations to POC flux magnitude and mechanisms will require robust, statistical relationships to be identified among ocean color, phytoplankton community composition, and carbon export flux. The two key taxa associated with POC flux in this study, diatoms and photosynthetic Hacrobia, are detectable using ocean color remote sensing by modeling phytoplankton pigment concentrations. This result demonstrates the potential of developing transfer functions to relate the information derived from ocean color satellites in the surface ocean to the relationships observed at depth between pigment-based phytoplankton community composition and POC flux magnitude. Further work is needed to extend the correlative relationships observed in this dataset over depths, space, and time, as well as integrating the prokaryotic phytoplankton community alongside the eukaryotic community examined here. The 18S rRNA gene was selected for this study to quantify the entire eukaryotic phytoplankton community at high taxonomic resolution, but the importance of cyanobacteria to carbon export has been well documented, particularly in salp fecal pellets [62–66]. In this dataset, we were confident in linking 18S rRNA gene relative sequence abundances to quantitative POC flux measurements, as 18S rRNA gene sequences for the major groups considered here were positively correlated with other metrics for phytoplankton community composition in the surface ocean (pigments, [imaging-in-] flow cytometry), but caution is warranted in extending these approaches to other ecosystems where these significant linear relationships between phytoplankton community metrics may vary [54].

Ocean color satellites view only the surface layer of the ocean, and the magnitude of sinking POC or the mechanisms by which it is exported are largely invisible from space. However, in this analysis, we demonstrated the potential to combine methods to reveal ecological mechanisms for phytoplankton export. In both ecosystems, we found that large particles were the key to carbon export, and that these particles exported distinct phytoplankton taxa from the surface ocean to depth. By developing a predictive ratio of pigment-based phytoplankton taxa shared between the surface and deep ocean to model POC flux magnitude in the mesopelagic, we showed that there are quantitative links between phytoplankton community composition, POC export flux magnitude, and mechanisms for export. This work starts to identify which information from the surface ocean is needed from satellites to predict POC flux dynamics in the mesopelagic. We suggest that diatoms and Hacrobia, which can be detected from surface ocean phytoplankton pigments and thus from PACE-resolution data, are particularly important. Since PACE launched in February 2024, the available information about surface ocean color and phytoplankton communities around the globe is rapidly increasing and improving. With this improved resolution to describe the surface ocean phytoplankton community, we will be able to extend the predictive model shown here to the surface ocean and test its performance across ecosystems.

## Supplementary Material

Kramer_etal_supporting_info_wraf105

## Data Availability

All DNA sequence data, POC flux data, and HPLC pigment data can be found on the EXPORTS page of NASA’s SeaWiFS Bio-optical Archive and Storage System (SeaBASS): https://seabass.gsfc.nasa.gov/experiment/EXPORTS. Bulk sediment trap and individual particle DNA data can be found at: https://seabass.gsfc.nasa.gov/archive/MBARI/durkin/EXPORTS. Whole seawater DNA data can be found at: https://seabass.gsfc.nasa.gov/archive/URI/rynearson/EXPORTS/. Bulk POC flux data can be found at: https://seabass.gsfc.nasa.gov/archive/MAINE/estapa/EXPORTS/. Code for DNA data analysis can be found at: https://github.com/sashajane19/EXPORTS_particle_DNA. If you are unable to find any data products or code, please contact the corresponding author.
