# Supplementary material for: Sinking particles exporting diatoms and Hacrobia predict the magnitude of oceanic POC flux
Source: ISME J. 2025 May 22;19(1):wraf105. doi: 10.1093/ismejo/wraf105 (PMC12145875; doi:10.1093/ismejo/wraf105)
Supplement: Kramer_etal_supporting_info_wraf105 [file kramer_etal_supporting_info_wraf105.pdf]

Supporting Information for

Sinking particles exporting diatoms and hacrobia predict the magnitude of oceanic POC flux

Sasha J. Kramer[1*], Erin L. Jones[2], Margaret L. Estapa[3], Nicola L. Paul[4], Tatiana A. Rynearson[2], Alyson E. Santoro[4], Sebastian Sudek[1], Colleen A. Durkin[1]

[1] Monterey Bay Aquarium Research Institute, Moss Landing CA USA
[2] Graduate School of Oceanography, University of Rhode Island, Narragansett RI USA
[3] School of Marine Sciences, Darling Marine Center, University of Maine, Walpole ME USA
[4] Department of Ecology, Evolution, and Marine Biology, University of California, Santa Barbara CA USA

*Corresponding author: Sasha J. Kramer, skramer@mbari.org

Competing Interests: The authors declare no competing interests.

This file includes:

Supplementary Methods
Supplementary References
Figures S1 to S6
Table S1 to S2

S1. Supplementary Methods:

S1.1 Sediment trap deployment and sample treatment:

Each trap platform had four collection tubes at each depth (diameter = 12.7 cm). Two of the tubes contained 0.3% formaldehyde-poisoned brine (70 ppt salinity) buffered to pH 8 with borate and overlain with 1 μm filtered surface seawater [1]. Samples from these tubes were used for measurements of POC and other bulk quantities. POC analysis is described in detail in [1]. Total particulate carbon was measured by combustion elemental analysis. Particulate inorganic carbon (PIC) was measured separately via coulometric titration [2] and POC fluxes were calculated by subtracting PIC from the total particulate carbon concentration. Uncertainty estimates were calculated from sample replicates.

The third tube contained either RNAlater [3] or 0.3% formalin and was used for bulk extraction of nucleic acids (see Section S1.2). A comparison of trap preservation methods (RNAlater vs. formalin) found that these samples had comparable Shannon diversity and eukaryotic community composition across methods, but bacterial community composition differed (N.L. Paul, *pers. comm.*). The fourth tube contained a polyacrylamide gel overlain by 1μm filtered surface seawater [4, 5] and was used for measurements of nucleic acids in individual particles [6] and for quantitative particle classification and imaging [7]. Upon trap recovery, particles collected in bulk collection tubes were allowed to settle for at least one hour before removing overlying seawater and draining the bottom sample layer. Samples were pre-filtered using a 330 μm mesh to separate zooplankton that swam actively into the sample. Zooplankton swimmers were visualized with a dissecting microscope and removed from the mesh (details in [4]), and the remaining particles were rinsed back into the sample using filtered seawater.

Measured POC fluxes in the North Atlantic surface tethered traps (STTs) in the third deployment may have been subject to hydrodynamic effects, with evidence for under-collecting in the upper traps ([8]; Margaret L. Estapa, *pers. comm.*). However, both the bulk POC and DNA samples would have been affected by these hydrodynamic effects, so these paired samples are still used here for comparison.

S1.2 18S rRNA gene amplification and sequence analysis

The V4 hypervariable region of the 18S rRNA gene was amplified using universal eukaryotic primers Reuk454FWD1 and V4r [9] on all sample types. Because seawater samples and particle samples were sequenced at different facilities, different Illumina-specific adaptors were added to the 5'ends of the primer used to amplify seawater DNA (forward5'- tcgtcggcatcagatgtgtataagagacagccagcascygcggtaattcc-3', reverse 5'-GTCTCGTGGGCTCGGAGATGTGTATAAGAGACAGACTTTCGTTCTTGAT-3') compared to the primers used to amplify particle DNA [ref 42] (forward 5'-acactgacgacatggttctacaccagcascycgcctaattcc -3' and reverse 5'-TACGGTAGCAGAGACTTGGTCTACTTTCGTTCTTGAT-3'). Seawater DNA was amplified in PCR reactions containing 1X AccuStart II PCR mix (VWR 89235-018), 0.3 μM each forward and reverse primer, and 20-100 ng DNA template using the following protocol: 2 min at 94°C, followed by 30 cycles of 94°C for 30 s, 55°C for 30 s, and 72°C for 1 min, and a final 10 min incubation at 72°C. Particle DNA was amplified in 25 μl PCR reactions using the KAPA Hifi Hotstart PCR kit (Roche KK2502) containing 1x buffer, 0.2 U polymerase, 0.3 mM dNTP, 0.3 mM each forward and reverse primer and 5 μl DNA extract with the following protocol: 5 min at 95°C, followed by 35 cycles 98°C for 30 sec, 62°C for 45 sec, and 1 min at 72°C.

PCR amplicons were cleaned with Ampure XP beads (Beckman Coulter, Brea, CA, USA) and quantified with the Qubit High Sensitivity DNA Assay Kit (ThermoFisher Scientific, Waltham, MA, USA). Amplicons from surface seawater samples were amplified for an additional five cycles to add Nextera indices and adaptors (Illumina, San Diego, CA, USA) and cleaned again with Ampure XP beads. These PCR products were pooled and quantified with the KAPA qPCR kit (Kapa Biosystems, Wilmington, MA, USA) prior to MiSeq (Illumina) sequencing with V3 chemistry (2 × 300 bp reads) at the University of Rhode Island Genomics and Sequencing Center. For sediment trap samples, barcoding and additional cleanup steps were performed at the Michigan State University Sequencing center prior to MiSeq (Illumina) sequencing with V3 chemistry (2 x 250 bp reads).

Extracting and amplifying DNA from three physically different sample types (bulk trap, surface seawater, and individual particles) required the use of three different DNA extraction protocols, each of

which may introduce distinct extraction biases. Though we could not avoid this methodological difference, we made efforts to make the datasets as comparable as possible. All extraction protocols included an initial bead beating or prolonged vortexing step. The same PCR primers were used for all samples. Although some samples were sequenced at different facilities, each sequencing run also included one mock community sample [10] to confirm that no bias in relative ASV community composition was introduced during the different sequencing runs (E.L. Jones, *pers. comm.*).

Supplementary References

1. Estapa M et al. Biogenic sinking particle fluxes and sediment trap collection efficiency at Ocean Station Papa. *Elem Sci Anthr* 2021;**9**:00122. https://doi.org/10.1525/elementa.2020.00122
2. Honjo S et al. Particle fluxes to the interior of the Southern Ocean in the Western Pacific sector along 170°W. *US South Ocean JGOFS Program AESOPS* 2000;**47**:3521–3548. https://doi.org/10.1016/S0967-0645(00)00077-1
3. Malmstrom R. RNAlater Recipe. 2015. https://dx.doi.org/10.17504/protocols.io.c56y9d
4. Durkin CA et al. A visual tour of carbon export by sinking particles. *Glob Biogeochem Cycles* 2021;**35**. https://doi.org/10.1029/2021GB006985
5. Durkin CA, Estapa ML, Buesseler KO. Observations of carbon export by small sinking particles in the upper mesopelagic. *Part Aquat Environ Invis Exopolymers Sink Aggreg* 2015;**175**:72–81. https://doi.org/10.1016/j.marchem.2015.02.011
6. Stephens BM et al. Direct observations of microbial community succession on sinking marine particles. *ISME J* 2024;**18**:wrad010. https://doi.org/10.1093/ismejo/wrad010
7. Amaral V, Durkin CA. A computer vision-based approach for estimating carbon fluxes from sinking particles in the ocean. *Limnol Oceanogr Methods* 2024. https://doi.org/10.1002/lom3.10665
8. Siegel D et al. Dynamics of aggregates and sinking carbon fluxes in a turbulent ocean. *EarthArXiv* 2024. https://doi.org/10.31223/X58709
9. Bradley IM et al. Design and evaluation of Illumina MiSeq-compatible, 18S rRNA gene-specific primers for improved characterization of mixed phototrophic communities. *Appl Environ Microbiol* 2016;**82**:5878–5891. https://doi.org/10.1128/AEM.01630-16
10. Catlett D et al. Evaluation of accuracy and precision in an amplicon sequencing workflow for marine protist communities. *Limnol Oceanogr Methods* 2020;**18**:20–40. https://doi.org/10.1002/lom3.10343

Supporting Figures

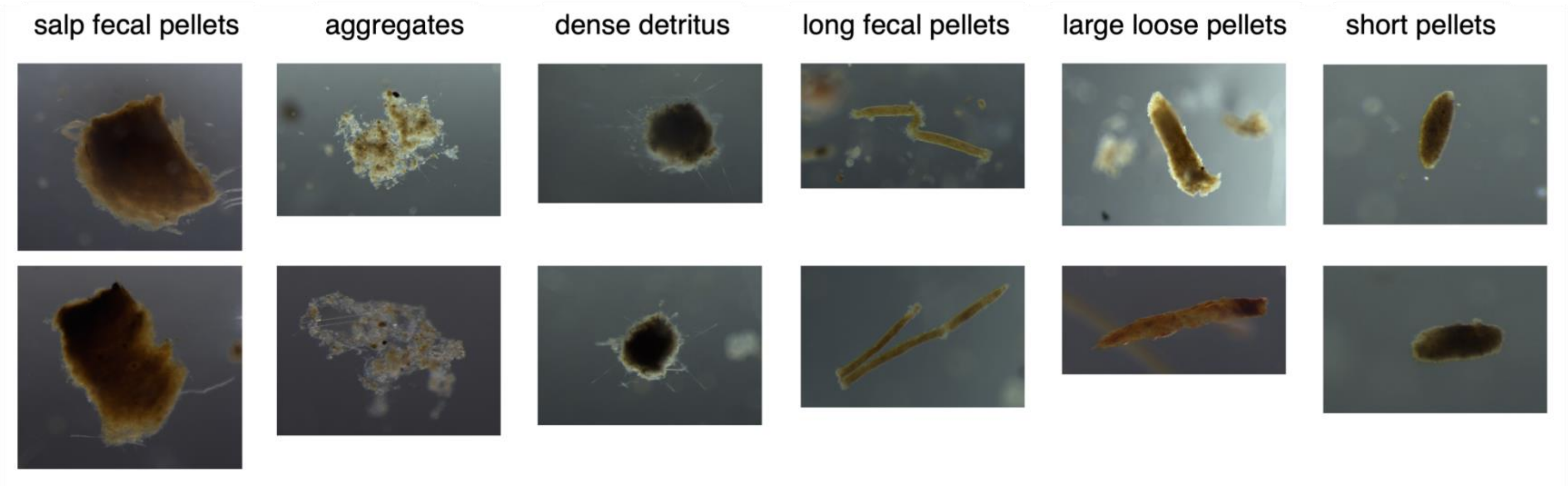

Figure S1. Example images for the major particle types described in this analysis.

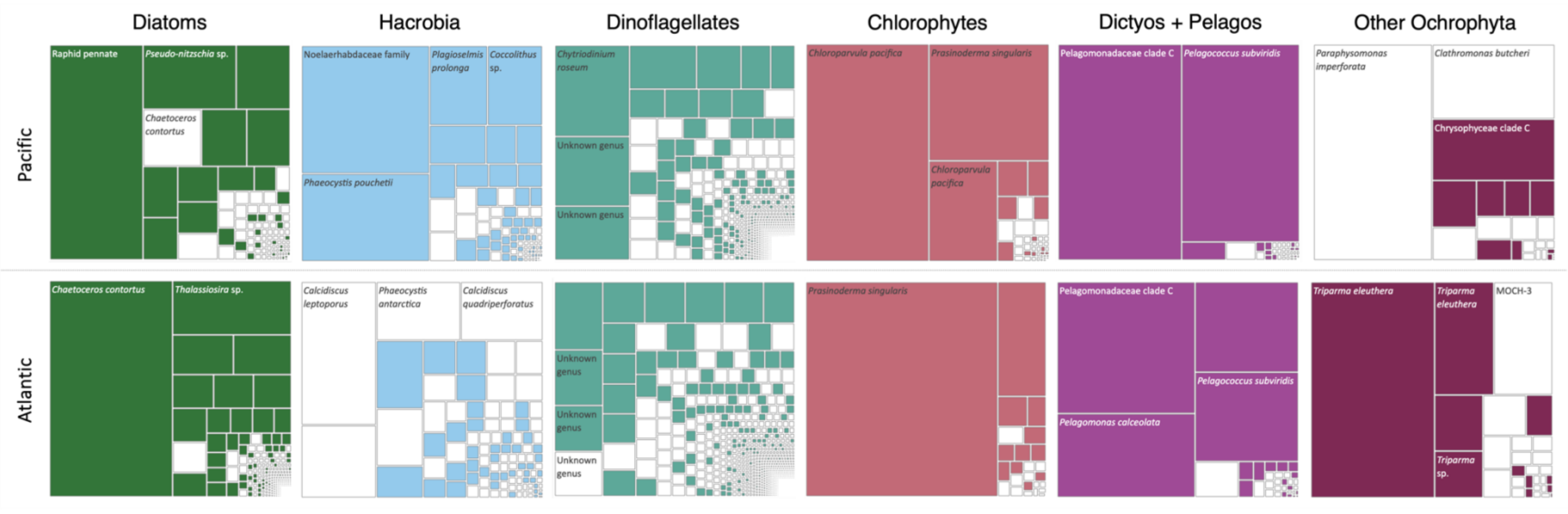

Figure S2. Phytoplankton 18S rRNA gene community composition within all sinking particle samples compared to those detected in the surface seawater. ASVs detected in bulk particles and individual particles were grouped by pigment-based phytoplankton class in the North Pacific (top row) and North Atlantic (bottom row). Each small box within a larger box represents an individual ASV within that group. If the box is colored in, that ASV was found in a surface seawater sample. Boxes that are not colored in represent ASVs that were not found in the surface.

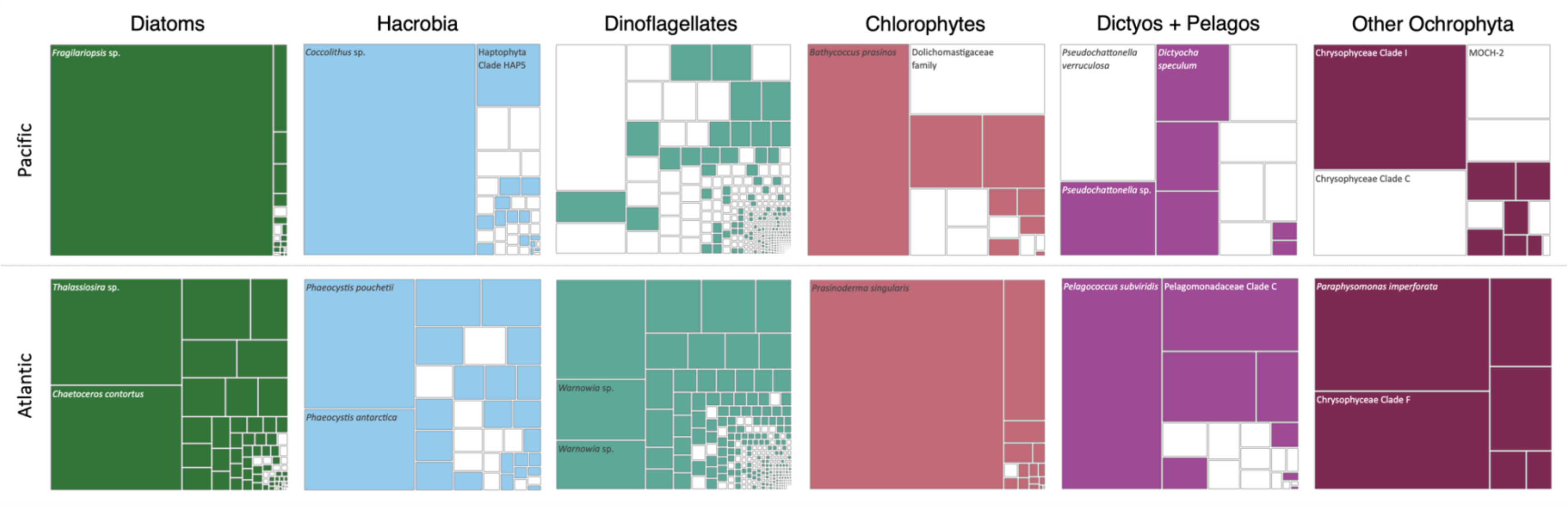

Figure S3. Phytoplankton 18S rRNA gene community composition within bulk-collected sinking particles compared to those detected in individual-isolated large particles. Phytoplankton ASV abundances were grouped by major pigment classes in bulk sinking particles in the North Pacific (top row) and North Atlantic (bottom row). Each small box within a larger box represents an individual ASV within that group. If the box is colored in, that ASV was also found in an individual particle sample. Boxes that are not colored in represent ASVs that were found in bulk sediment trap samples but not in individual particle samples.

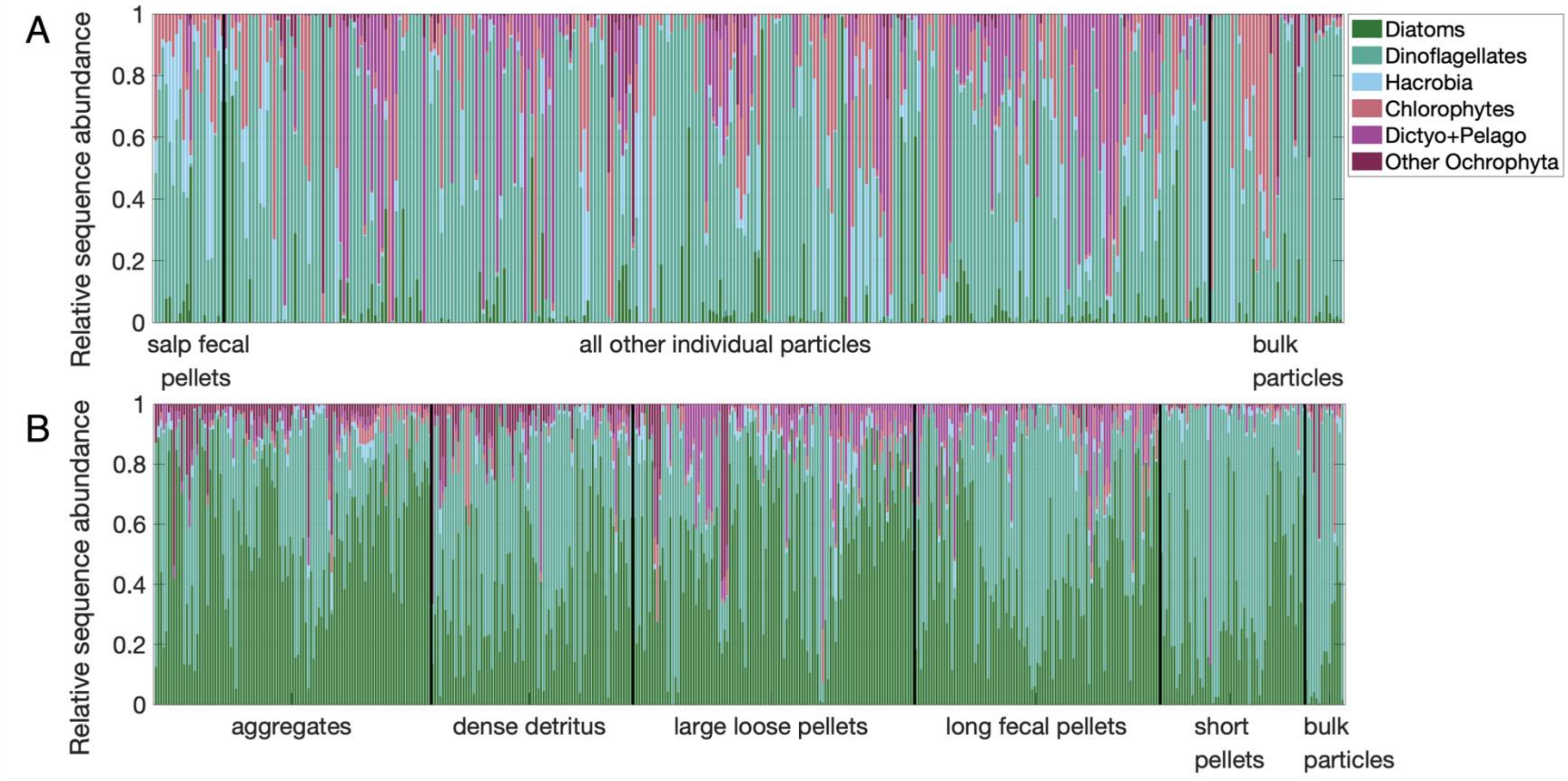

Figure S4. Phytoplankton community composition within every particle samples. The relative 18S rRNA gene sequence abundances were grouped into six major pigment-based phytoplankton groups. Vertical black lines separate statistically different particle types in (A) the North Pacific and (B) the North Atlantic. Statistical differences in community composition were determined at the level of ASVs rather than the grouped taxonomic data shown here.

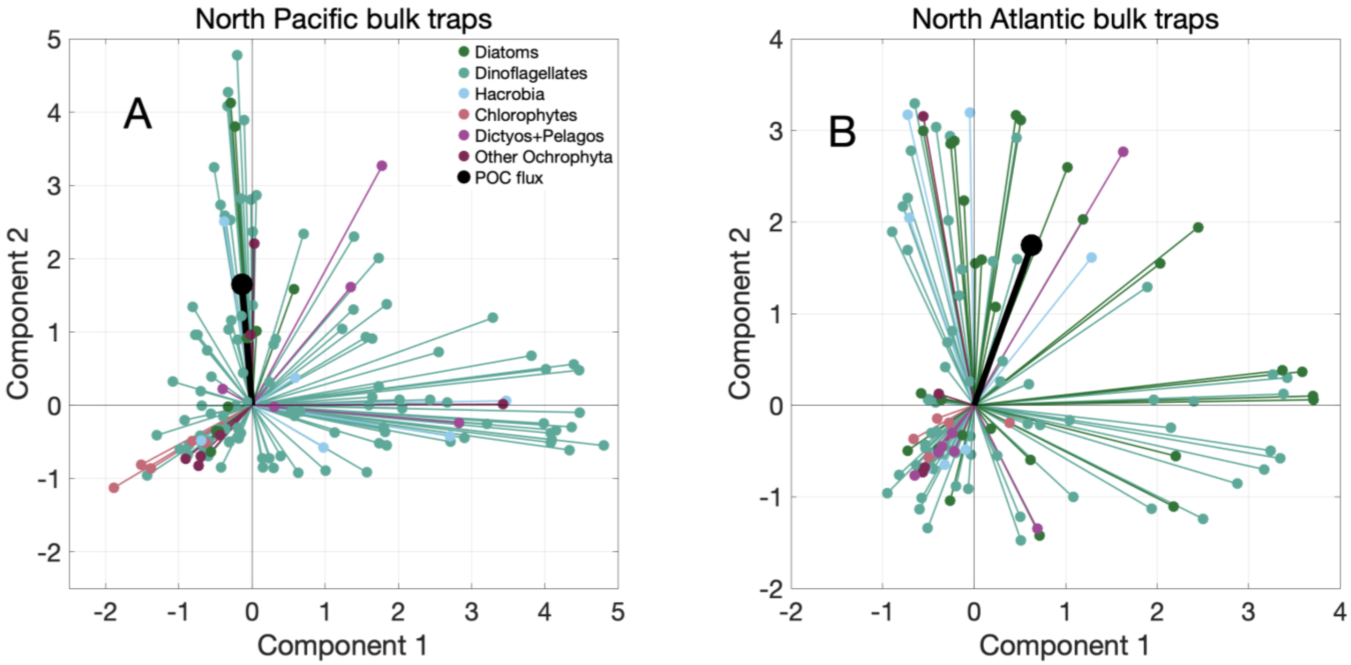

Figure S5. Relationship between phytoplankton ASV relative abundances and particulate organic carbon (POC) fluxes. Principal components analysis showing the first two components for all bulk trap ASVs and POC flux magnitude in (A) the North Pacific and (B) the North Atlantic. ASV loadings are colored based on their broad pigment-based phytoplankton group and POC flux is in black.

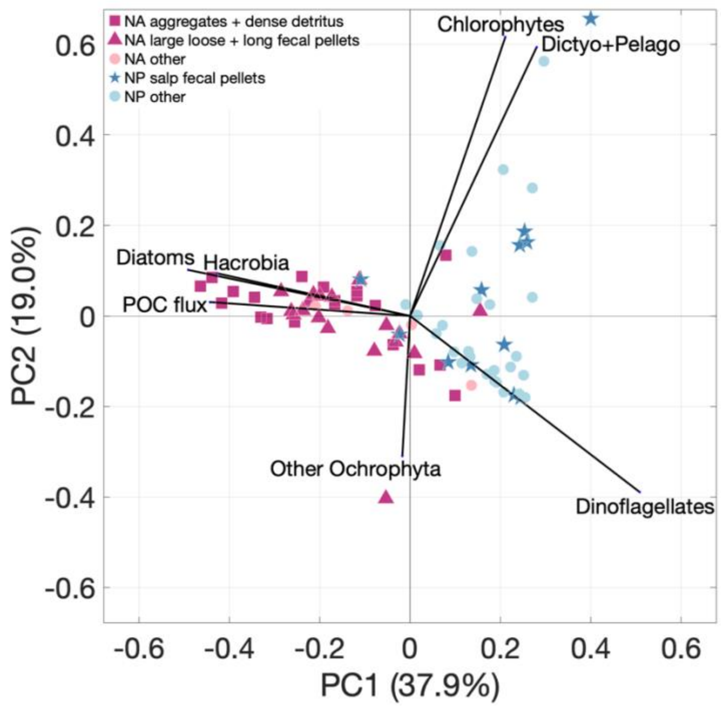

Figure S6. Covariance of POC flux by individual particle types with the 18S rRNA gene phytoplankton community within those particles. Principal components analysis (PCA) was performed on particle-specific data. The orientation of each group of particles from each trap and each PCA input variable (black lines) is shown for the first 2 components (Component 1 = 37.9% of variability; Component 2 = 19% of variability).

Table S1. List of photosynthetic taxa used in this analysis.

Kingdom	Supergroup	Division	Class
Eukaryota	Alveolata	Dinoflagellata	Dinophyceae
Eukaryota	Archaeplastida	Chlorophyta	Palmophyllophyceae
Eukaryota	Archaeplastida	Chlorophyta	Mamiellophyceae
Eukaryota	Stramenopiles	Ochrophyta	Pelagophyceae
Eukaryota	Archaeplastida	Streptophyta	Embryophyceae
Eukaryota	Archaeplastida	Chlorophyta	Chloropicophyceae
Eukaryota	Stramenopiles	Ochrophyta	Bacillariophyta
Eukaryota	Hacrobia	Haptophyta	Prymnesiophyceae
Eukaryota	Alveolata	Dinoflagellata	Dinophyta_X
Eukaryota	Stramenopiles	Ochrophyta	Dictyochophyceae
Eukaryota	Alveolata	Dinoflagellata	NaN
Eukaryota	Hacrobia	Cryptophyta	Cryptophyceae
Eukaryota	Stramenopiles	Ochrophyta	NaN
Eukaryota	Stramenopiles	Ochrophyta	MOCH-2
Eukaryota	Archaeplastida	Chlorophyta	Prasino-Clade-9
Eukaryota	Stramenopiles	Ochrophyta	Chrysophyceae
Eukaryota	Stramenopiles	Ochrophyta	MOCH-5
Eukaryota	Stramenopiles	Ochrophyta	Bolidophyceae
Eukaryota	Alveolata	Dinoflagellata	Noctilucophyceae
Eukaryota	Hacrobia	Haptophyta	Haptophyta_X
Eukaryota	Archaeplastida	Chlorophyta	Pyramimonadales
Eukaryota	Stramenopiles	Ochrophyta	Phaeophyceae
Eukaryota	Stramenopiles	Ochrophyta	MOCH-3
Eukaryota	Archaeplastida	Chlorophyta	NaN
Eukaryota	Stramenopiles	Ochrophyta	MOCH-4
Eukaryota	Archaeplastida	NaN	NaN
Eukaryota	Archaeplastida	Chlorophyta	Prasino-Clade-V
Eukaryota	Hacrobia	Haptophyta	NaN
Eukaryota	Hacrobia	Haptophyta	Haptophyta_Clade_HAP2
Eukaryota	Rhizaria	Cercozoa	Chlorarachniophyceae
Eukaryota	Hacrobia	Haptophyta	Haptophyta_Clade_HAP3
Eukaryota	Archaeplastida	Chlorophyta	Chlorodendrophyceae
Eukaryota	Stramenopiles	Ochrophyta	Raphidophyceae
Eukaryota	Excavata	Discoba	Euglenozoa
Eukaryota	Hacrobia	Haptophyta	Haptophyta_Clade_HAP5
Eukaryota	Hacrobia	Haptophyta	Haptophyta_Clade_HAP4

Kingdom	Supergroup	Division	Class
Eukaryota	Archaeplastida	Chlorophyta	Chlorophyceae
Eukaryota	Alveolata	Dinoflagellata	Ellobiophyceae
Eukaryota	Archaeplastida	Chlorophyta	Ulvophyceae
Eukaryota	Archaeplastida	Chlorophyta	Chlorophyta_X
Eukaryota	Archaeplastida	Streptophyta	NaN
Eukaryota:plas	Stramenopiles:plas	Ochrophyta:plas	Bacillariophyta:plas
Eukaryota:nucl	Hacrobia:nucl	Cryptophyta:nucl	Cryptophyceae:nucl
Eukaryota	Archaeplastida	Chlorophyta	Pyramimonadophyceae
Eukaryota	Archaeplastida	Prasinodermophyta	Prasinodermophyceae
Eukaryota	Archaeplastida	Chlorophyta	Prasino-Clade-VIII
Eukaryota	Archaeplastida	Chlorophyta	Trebouxiophyceae
Eukaryota	Hacrobia	Haptophyta	Rappephyceae
Eukaryota:plas	Archaeplastida:plas	Chlorophyta:plas	NaN
Eukaryota:plas	Excavata:plas	Discoba:plas	Euglenida:plas
Eukaryota	Hacrobia	Haptophyta	Rappephyceae
Eukaryota:plas	Archaeplastida:plas	Chlorophyta:plas	Chloropicophyceae:plas
Eukaryota	Stramenopiles	Ochrophyta	Ochrophyta_X

Table S2. Relationship between phytoplankton taxa (relative to all other summed phytoplankton groups) and POC flux for the North Pacific (NP), North Atlantic (NA), and the summed EXPORTS dataset (all). Insignificant *P* values are highlighted in red.

Input data	NP linear fit	NP R^2 and *P* value	NA linear fit	NA R^2 and *P* value	All linear fit	All R^2 and *P* value
Diatoms	y = -1.3x + 1.1	0.04 0.31	y = -1.6x + 6.0	0.08 0.30	y = 1.9x + 1.9	0.10 0.32
Hacrobia	y = 1.2x + 0.88	0.05 0.26	y = 4.9x + 3.2	0.57 0.001	y = 6.2x + 1.2	0.54 <<0.001
Diatoms + Hacrobia	y = 0.17x + 0.97	0.002 0.83	y = 3.4x + 0.24	0.48 0.003	y = 3.4x + 0.47	0.72 <<0.001
